# Structural disorder and transformation in crystal growth: direct observation of ring-opening isomerization in a metal–organic solid solution

**DOI:** 10.1107/S2052252514015966

**Published:** 2014-08-20

**Authors:** Ji-Jun Jiang, Jian-Rong He, Xing-Qiang Lü, Da-Wei Wang, Guo-Bi Li, Cheng-Yong Su

**Affiliations:** a MOE Laboratory of Bioinorganic and Synthetic Chemistry, State Key Laboratory of Optoelectronic Materials and Technologies, Lehn Institute of Functional Materials, School of Chemistry and Chemical Engineering, Sun Yet-Sun University, Guangzhou 510275, People’s Republic of China; b State Key Laboratory of Organometallic Chemistry, Shanghai Institute of Organic Chemistry, Chinese Academy of Sciences, Shanghai 200032, People’s Republic of China

**Keywords:** crystallization, structural transformation, ring-opening isomerism, solid solution, disorder

## Abstract

A rare example is reported in which discrete Ag_2_ *L*_2_ ring and (Ag*L*)_∞_ chain motifs [*L* = *N*,*N*′-bis(3-imidazol-1-yl-propyl)-pyromellitic diimide] co-crystallize in the same crystal lattice with varying ratios and degrees of disorder. Crystal structures obtained from representative crystals reveal compatible packing arrangements of the cyclic and polymeric isomers within the crystal lattice, which enables them to co-exist within a crystalline solid solution. A feasible pathway for transformation between the isomers is suggested *via* facile rotation of the coordinating imidazolyl groups. This chemical system could provide a chance for direct observation of ring-opening isomerization at the crystal surface. Mass spectrometry and ^1^H NMR titration show a dynamic equilibrium between cyclic and oligomeric species in solution, and a potential crystallization process is suggested involving alignment of precursors directed by aromatic stacking interactions between pyromellitic diimide units, followed by ring-opening isomerization at the interface between the solid and the solution. Both cyclic and oligomeric species can act as precursors, with interconversion between them being facile due to a low energy barrier for rotation of the imidazole rings. Thermogravimetric analysis and variable-temperature powder X-ray diffraction indicate a transition to a different crystalline phase around 120°C, which is associated with loss of solvent from the crystal lattice.

## Abstract 2

The co-crystallization of cyclic and polymeric isomers in the same crystal in varying ratios with the skeleton frameworks packed in a geometrically compatible and energetically similar fashion gives a chance to rationalize ring-opening isomerization in a crystal growth process.

## 1. Introduction   

The application of design principles that are based on the concepts of self-assembly has over the past decades produced diverse functional crystalline solids possessing important physicochemical properties (*e.g.* Lehn, 2002 ▶; Whitesides & Boncheva, 2002 ▶; Janiak, 2003 ▶; Mathias & Stoddart, 1992 ▶; Cook *et al.*, 2013 ▶). Applying these principles to coordination chemistry has also offered chemists opportunities to control the chemical and structural nature of coordination assemblies and, therefore, their functionalities. Two rapidly advancing topics in the field of metal–organic materials (MOMs) include: (1) crystal engineering of metal–organic frameworks (MOFs) or coordination polymers (CPs) that feature in infinite and periodic composite organic/inorganic frameworks (*e.g.* Kitagawa *et al.*, 2004 ▶; Khlobystov *et al.*, 2001 ▶; Hagrman *et al.*, 1999 ▶; Batten & Robson, 1998 ▶; Hosseini, 2005 ▶; Ockwig *et al.*, 2005 ▶; Wang *et al.*, 2013 ▶), and (2) molecular engineering of supramolecular ensembles, such as coordination cages, rings, helicates *etc.* that are characteristic of finite and discrete structures with well defined shape, size and topology (*e.g.* Leininger *et al.*, 2000 ▶; Fujita *et al.*, 2001 ▶; Swiegers & Malefetse, 2000 ▶; Caulder & Raymond, 1999 ▶; Saalfrank *et al.*, 2000 ▶; Turner *et al.*, 2004 ▶; Ronson *et al.*, 2013 ▶; Chen *et al.*, 2007 ▶).

Despite increasing interest in these two promising topics, surprisingly little attention has been paid to the inherent relationship between the finite and infinite systems, although they both obey similar synthetic self-assembly strategies and examples of closely related cyclic and polymeric structures obtained individually from the same metal ions and ligands are known in the literature (*e.g.* Lozano *et al.*, 2001 ▶; Brandys & Puddephatt, 2001 ▶; Qin *et al.*, 2002 ▶; Puddephatt, 2008 ▶; Liang *et al.*, 2009 ▶). Insight into this interdisciplinary area between the discrete and extended structures may be able to shed light on the understanding of crystal engineering, in which the growth mechanisms of the periodic crystalline solids of MOFs and CPs remain largely unknown, probably due to their typical insolubility in solvents and the difficulty in determining the degree of polymerization both in solution and in crystal growth. On the contrary, most of the discrete coordination assemblies are detectable in solution. One of the efforts to bridge the finite and infinite analogues has been made by Puddephatt and James, who introduced the term *ring-opening polymerization* (ROP) to explain the formation of extended structures from discrete cyclic molecular precursors (*e.g.* Fromm *et al.*, 2005 ▶; Miller *et al.*, 2004 ▶; Brandys & Puddephatt, 2002 ▶; Shin *et al.*, 2003 ▶; Abourahma *et al.*, 2002 ▶; Lin & Yip, 2006 ▶). It is believed that a dynamic equilibrium exists between the discrete species and ring-opened oligomers in solution, while the extended coordination polymers form only on the surface of the growing crystals (*e.g.* Su, Cai *et al.*, 2003 ▶; Chen *et al.*, 2005 ▶; Burchell *et al.*, 2003 ▶; Paulisse & Sjibesma, 2003 ▶). To look for evidence to support this hypothesis, James offered the challenge to ‘*get evidence of ROP at the crystal surface*’ (James, 2004 ▶).

More generally, structural diversification is frequently considered in relation to assembly processes and crystal engineering. Moulton & Zaworotko (2001 ▶) proposed the concept of *supramolecular isomerism* to describe the structural diversity in the realm of CPs, which establishes a way of systematizing and classifying the structures of CPs consisting of identical building blocks in the same ratio but having different crystal structures. Various types of supramolecular isomerism, such as *structural isomerism* (Hennigar *et al.*, 1997 ▶), *conformational isomerism* (MacGillivray *et al.*, 2001 ▶) and *topological isomerism* (*e. g.* Blake *et al.*, 2001 ▶; Gao *et al.*, 2002 ▶), have been reported. In a few cases, mixed isomers co-exist in the same crystal (*e.g.* Carlucci *et al.*, 2004 ▶; Li *et al.*, 2005 ▶). To classify supramolecular isomers displaying distinct features of cyclic and polymeric structures, we introduced the term *ring-opening isomerism* (ROI), which represents a common type of supramolecular isomerism in which the isomers are structurally related to each other by at least one ring-opening transformation (*e.g.* Su, Goforth *et al.*, 2003 ▶; Zhang *et al.*, 2010 ▶). ROI can be considered as a structural diversification phenomenon produced *via* the above-mentioned ROP process.

Previously, we have investigated the dynamics of a series of disilver(I) metallacycles in solution and found that the metal–ligand exchange energy barrier is small and roughly comparable to that of moderate hydrogen bonding (Chen *et al.*, 2005 ▶). This result explains why the ROI phenomenon can readily occur in solution, but direct evidence of the ROP process in crystallization is still absent. In this paper we present an interesting example where the ROI phenomenon is observed directly in metal–organic solid solutions, in which both discrete *M*_2_ *L*_2_ rings and polymeric (*ML*)_∞_ chains co-crystallize in the same crystal lattice with varying ratios and degrees of order/disorder. The specific packing fashion of the discrete rings and the polymeric chains enables us to understand how the ROP process takes place during crystallization, thus providing a potential prototypical model to demonstrate the ROI phenomenon in crystal growth.

## 2. Experimental   

### 2.1. Materials and methods   

**Figure 1:**
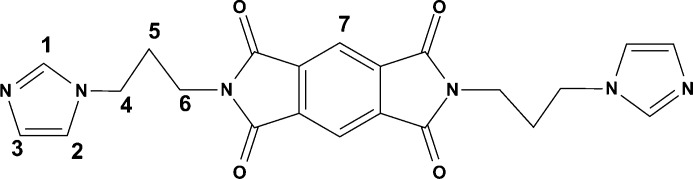
Molecular structure of the ligand *L*.

All chemicals were purchased from commercial sources and used without further purification. *N*,*N*′-Bis(3-imidazol-1-yl-propyl)-pyromellitic diimide (*L*, see Fig. 1 ▶) was prepared according to the previously described method (Lü *et al.*, 2006 ▶). Elemental analyses were carried out on a Perkin–Elmer 240 elemental analyser. IR spectra were obtained on a Bruker EQUINOX55 FT–IR spectrophotometer using KBr discs in the 4000–400 cm^−1^ region. ^1^H NMR measurements were carried out on INOVA 500NB or Mercury-Plus 300 spectrometers with SiMe_4_ as internal standard. The powder XRD patterns were recorded on a D/Max-IIIA diffractometer with Cu *K*α radiation (λ = 1.5406 Å) at a scanning rate of 1° (2θ) min^−1^. Thermogravimetric analysis (TGA) was performed in air at a heating rate of 10°C min^−1^ on a NETZSCH Thermo Microbalance TG 209 F3 Tarsus. The excitation and emission spectra were obtained on a HITACHI 850 spectrometer.

### 2.2. Synthesis of complexes   

Complexes {[Ag*L*(CF_3_CO_2_)]·solvent}_*n*_ (**1**·solvent) in different polymorphic forms were prepared by a general method with varying crystallization times. A solution of AgCF_3_CO_2_ (13 mg, 0.025 mmol) in tetrahydrofuran (THF; 5 ml) was layered carefully onto a solution of *L* (22 mg, 0.05 mmol) in CH_2_Cl_2_ (10 ml) in a test tube. The solutions were left to stand for a few days at room temperature, giving gradual growth of colourless block crystals. Yield: ∼ 54%. IR (KBr, cm^−1^): 3474w, 3111w, 2944w, 1777m, 1718vs, 1516w, 1456w, 1435w, 1397s, 1362m, 1297w, 1278w, 1238w, 1199m, 1173m, 1130s, 1043w, 1026w, 918w, 883w, 861w, 828w, 799w, 756w, 725m, 657w, 627w, 591w, 559w.

### 2.3. X-ray crystallography   

Several crystals were randomly selected from different crystallization batches and mounted on glass fibres for structural determination, offering two apparent polymorphic forms **A** (*Pbcm*) and **B** (*Ibam*). All diffraction data were collected on a Bruker Smart 1000 CCD diffractometer with graphite-monochromated Mo *K*α radiation (λ = 0.7107 Å) at room temperature using the program *SMART* and processed by *SAINT-Plus* (Bruker, 1998 ▶). Absorption corrections were applied using *SADABS* (Bruker, 1998 ▶) and the structures were solved by direct methods and refined using full-matrix least-squares against *F* ^2^ using *SHELXTL* (Sheldrick, 2008 ▶). Non-H atoms were refined anisotropically, while H atoms were introduced in calculated positions and refined with fixed geometry with respect to their carrier atoms. For form **A**, due to poor crystal quality and severe disorder of the anions and solvent molecules, the *SQUEEZE* procedure (van der Sluis & Spek, 1990 ▶) implemented *via* the *WinGX* suite (Farrugia, 2012 ▶) was applied to account for regions of diffuse electron density that could not be satisfactorily modeled. The coordination frameworks generally display well defined positions; however, disorder of the Ag atoms and methylene imidazole donors is evident. Refinement of the site occupancies of the disordered Ag atoms indicates an 18/82% site distribution, while the disordered methylene imidazole fraction could not be satisfactorily modelled. Some of the C and N atoms exhibiting unusual displacement parameters were refined with SIMU/ISOR restraints in *SHELXTL*. For form **B**, the Ag atoms and methylene imidazole donors display statistical disorder over two positions with 50:50% site occupancy. The CF_3_CO_2_^−^ anions are disordered over two *C*_2_ symmetry-related positions, and modelled using DFIX/SADI restraints. All water solvent molecules were treated with partial occupancy and H atoms were not added. Both the anions and solvents were refined with DELU/SIMU/ISOR restraints. Crystallographic information for data collection and structural refinement is listed in Table 1 ▶, and selected bond lengths and angles are listed in Table S1.

## 3. Results and discussion   

### 3.1. Synthesis and X-ray diffraction   

**Figure 2:**
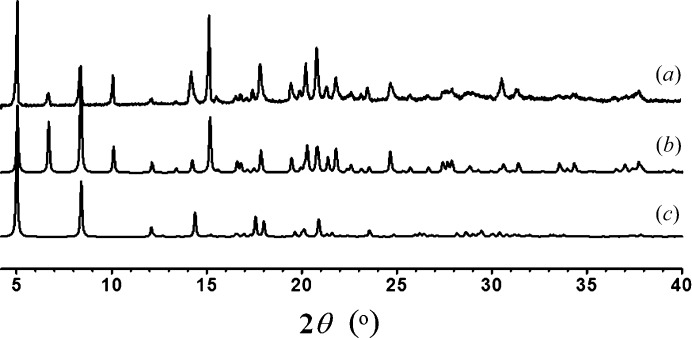
Comparison of powder X-ray diffraction patterns: (*a*) as-synthesized; (*b*) simulation based on the refined structure of form **B**; (*c*) simulation based on the refined structure of form **A**. Note that the refined structure of form **A** does not contain the disordered anions or solvent molecules, so the simulated diffraction pattern is only indicative.

The long ditopic, semi-flexible ligand containing a rigid three-fused-ring central base and two imidazole coordinating donors linked by two flexible propylene chains, *N*,*N*′-bis(3-imidazol-1-yl-propyl)-pyromellitic diimide (*L*, see Fig. 1 ▶), was prepared by reaction of pyromellitic dianhydride with 2 molar equivalents of *N*-3-aminopropyl-imidazole in DMF. The reaction of *L* with AgCF_3_CO_2_ in the presence of CH_2_Cl_2_ afforded complexes of the general formula {[Ag*L*(CF_3_CO_2_)]·solvent}*_n_* (**1**·solvent). The IR spectrum of the fresh sample indicated the presence of water molecules and CF_3_CO_2_^−^ anions in the crystals (Fig. S1), while ^1^H NMR measurements suggested inclusion of CH_2_Cl_2_ solvent molecules. The relative quantities of the solvents in the crystal were estimated by thermogravimetric analysis (TGA) to be two H_2_O and one CH_2_Cl_2_ molecule per [Ag*L*(CF_3_CO_2_)]_2_ unit (Fig. S2). The phase purity of the bulk sample was checked by comparing the measured powder X-ray diffraction (XRD) pattern with the simulations based on the single-crystal structure data (Fig. 2 ▶, *see below*). Solid-state photoluminescent measurements on the pure ligand and complex **1** indicated that the ligand-based emission was strengthened after coordination but keeping a similar peak profile. The emission maximum was red-shifted by 29 nm from 453 nm in the ligand to 482 nm in the complex. These findings suggest that the emission nature of the complex is basically ligand-centred (LC) but may contain a little ligand-to-metal charger-transfer (LMCT) contribution because of the presence of short Ag⋯Ag contacts (*see below*; Barbieri *et al.*, 2008 ▶).

Colourless crystals were readily available from a layered THF–CH_2_Cl_2_ solvent system in test tubes. Single-crystal X-ray diffraction screening of randomly selected crystals revealed that most of them, although appearing to be optically perfect, had apparently poor crystallinity. Careful structural analyses of a number of single crystals indicated the existence of two main polymorphic forms (Table 1 ▶): orthorhombic *Pbcm* (assigned as form **A**) and *Ibam* (assigned as form **B**). Most crystals probably crystallize in intermediate phases between these two forms, with varying ratios and degrees of disorder depending on the growth conditions. The difficulties are also reflected in the powder XRD patterns of the bulk sample. As seen from Fig. 2 ▶, the measured XRD pattern matches well with that simulated from the disordered form **B** (space group *Ibam*). The simulated XRD pattern based on form **A** (space group *Pbcm*) is only indicative due to omission of the solvent and anions, but all of the observed diffraction peaks also fit well with those of the measured sample. This indicates that the disorder details cannot directly be distinguished from the powder XRD data.

The chemical nature of the two crystal forms **A** and **B** can be interpreted as follows. For form **B** (*Ibam*), keeping only one or the other Ag atom site from the statistically disordered pair generates ordered crystal structures (both in space group *Ibam*) comprising only Ag_2_ *L*_2_ rings or only polymeric (Ag*L*)_∞_ chains, respectively. Idealized CIFs corresponding to these structures (*rings_Ibam.cif* and *chains_Ibam.cif*) are included in the supporting information for reference. The statistically disordered arrangement of the Ag sites in form **B** indicates that the two isomers form a solid solution. For form **A** (*Pbcm*), considering only the majority (82%) positions for the Ag atoms reveals a structure in which discrete Ag_2_ *L*_2_ rings and polymeric (Ag*L*)_∞_ chains alternate along the crystal *a* axis. An idealized CIF for this situation (*both_Pbcm.cif*) is also included in the supporting information. The presence of the second Ag atom site (18% site occupancy) indicates that the structure is part-way between the idealized form **A** structure and the form **B** solid solution. Thus, three idealized structures can be envisaged (all rings, all chains, alternating rings/chains along the *a* axis), and the actual crystals generally adopt structures partway between these.

**Figure 3:**
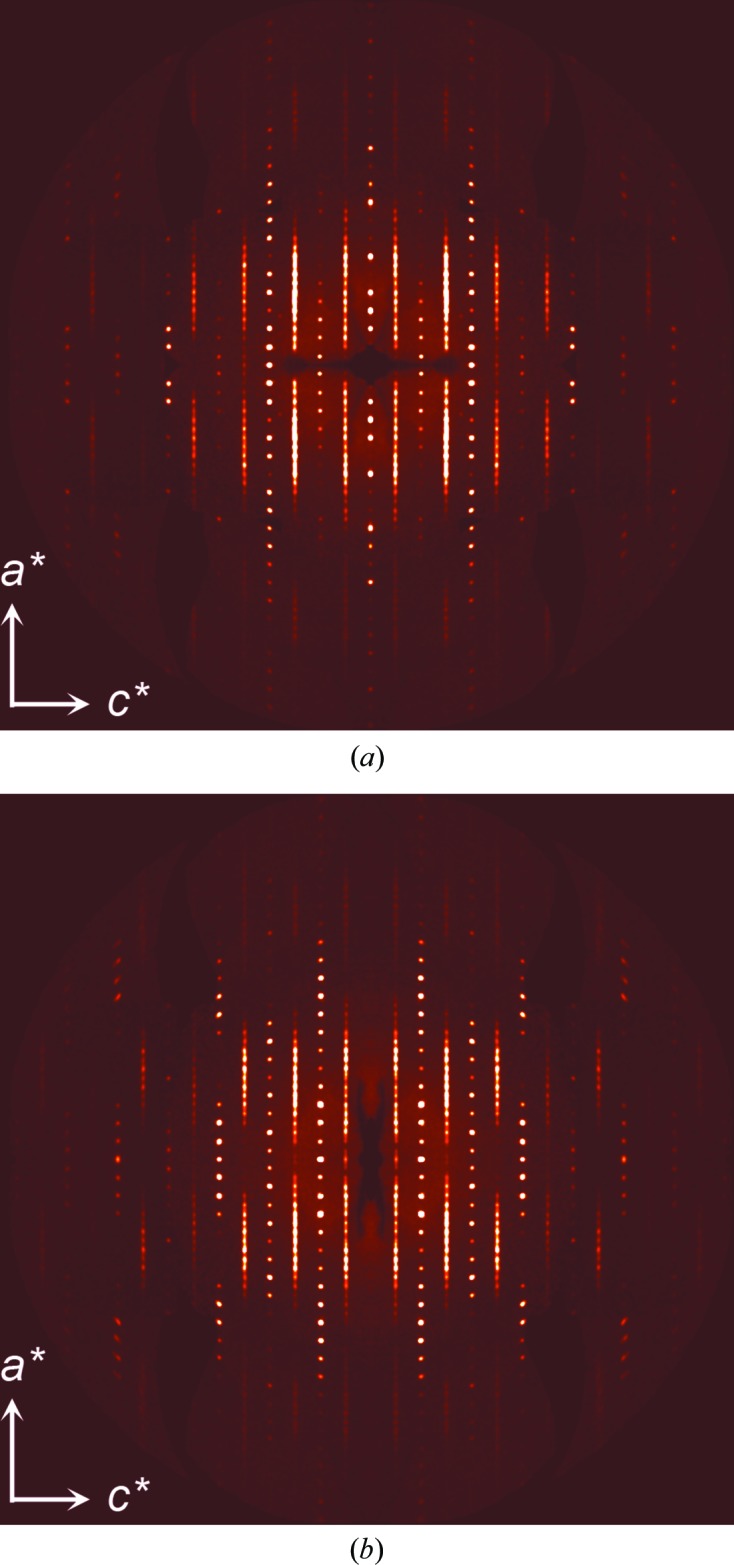
Simulated precession images for the *h*0*l* (*a*) and *h*1*l* (*b*) planes for a randomly selected crystal. Diffuse streaks along a* for odd values of *l* reveal one-dimensional disorder as described in the text.

To examine further the crystallographic origin of the problematic structural analyses, precession images in the a*c* planes of reciprocal space were reconstructed with a thickness of one pixel from the raw CCD area detector images using the *CrystAlisPro* program (Agilent, 2014 ▶). As shown in Fig. 3 ▶, diffuse streaks along a* are obvious in the reconstructed *h*0*l* and *h*1*l* layers for odd values of *l*, lying between rows of sharp Bragg reflections for even values of *l*. This indicates that the crystals exhibit one-dimensional disorder along the crystallographic *a* axis, consistent with an order/disorder type description of the structures (Moulton & Zaworotko, 2001 ▶; Bürgi *et al.*, 2001 ▶). More detailed examination of the disorder has not been undertaken for this study; the presented interpretation of the crystallographic data is sufficient to describe the observed chemical phenomenon.

### 3.2. Crystal packing and ring-opening isomerism   

**Figure 4:**
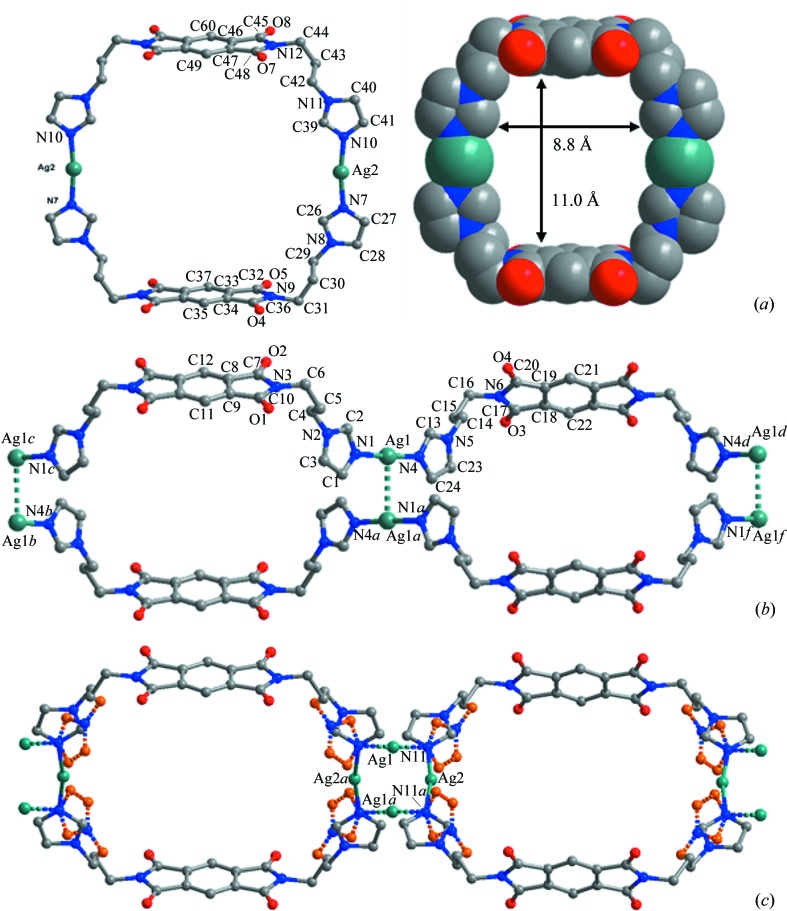
Structural motifs in the crystal structures of complex **1**: (*a*) Ag_2_ *L*_2_ ring and its space-filling model extracted from the (idealized) structure of form **A**; (*b*) twisted (Ag*L*)_∞_ double chains extracted also from form **A** (dotted lines indicate Ag⋯Ag contacts); (*c*) Ag_2_ *L*_2_ rings and (Ag*L*)_∞_ double chains co-existing in a disordered fashion in form **B**. Differently orientated methylene imidazole donors are distinguished by the solid and dotted bonds as well as C atoms in different colours.

As shown in Figs. 4 ▶(*a*) and (*b*), a crystal with the (idealized) structure of form **A** consists of two clearly distinguishable structural motifs. One is the discrete Ag_2_ *L*_2_ ring and the other is the polymeric (Ag*L*)_∞_ chain. The ring motif comprises two *L* ligands and two Ag atoms making up a large [Ag_2_ *L*_2_]^2+^ rectangle with Ag—N bond distances of 2.121 (9) and 2.124 (8) Å, and an N—Ag—N angle of 171.7 (3)°. The polymeric chain comprises [Ag*L*]^+^ repeating units that extend along the *c* axis with Ag—N bond distances of 2.053 (8) and 2.087 (10) Å, and with an N—Ag—N angle of 176.6 (4)°. There are strong argentophilic interactions between pairs of adjacent chains due to the short Ag⋯Ag contact [3.0776 (17) Å, significantly shorter than the sum of the van der Waals radii for two Ag atoms, 3.44 Å]. These Ag⋯Ag contacts link pairs of chains to form a twisted double-chain structure, exhibiting [(Ag_1/2_)_4_ *L*_2_]^2+^ rectangular subunits sharing two Ag atoms on each side. Such [(Ag_1/2_)_4_ *L*_2_]^2+^ metallacycles show rather similar shape to the [Ag_2_ *L*_2_]^2+^ ring motifs (Fig. 4 ▶ and S4), and display similar interior cavity dimensions (8 × 11 Å^2^). The difference is the orientation of the methylene imidazole donors accompanying the shift of the Ag metal centres.

Although the crystal quality of form **A** did not permit clear identification of the disordered methylene imidazole part, free refinement of the Ag site occupancy suggests a minor occupancy (18%) disorder component. This implies that, over the whole crystal lattice, the positions mainly occupied by Ag_2_ *L*_2_ rings are superimposed by 18% of (Ag*L*)_∞_ chains, and *vice versa*. Such structural disorder and the compatibility of the ring and chain isomers becomes even more clear in the structure of form **B**. As shown in Fig. 4 ▶(*c*), two methylene imidazole donors in each *L* ligand can adopt two orientations binding two different Ag atoms with statistical (50:50) occupancy. Therefore, formation of the discrete Ag_2_ *L*_2_ rings and the polymeric (Ag*L*)_∞_ chains appears to have equal probability and can be envisaged to occur in any crystal growth situation. Specifically, the [Ag_2_ *L*_2_]^2+^ ring has an Ag—N bond distance of 2.134 (7) Å and an N—Ag—N angle of 178.2 (4)°, while the [Ag*L*]^+^ repeating unit in the polymeric chain has an Ag—N bond distance of 2.130 (7) Å and an N—Ag—N angle of 167.8 (4)°. The argentophilic Ag⋯Ag contact is also formed between adjacent chains [3.086 (3) Å]. The disordered site distribution of Ag metal centres and the methylene imidazole donors makes clear that either the Ag_2_ *L*_2_ ring or (Ag*L*)_∞_ chains can be formed and can interconvert through rotation of the imidazole rings while retaining the same *syn* conformation of the pyromellitic diimide spacers. No matter which of the isomeric structural motifs is formed, the overall coordination skeleton frameworks display the same rectangular shape.

**Figure 5:**
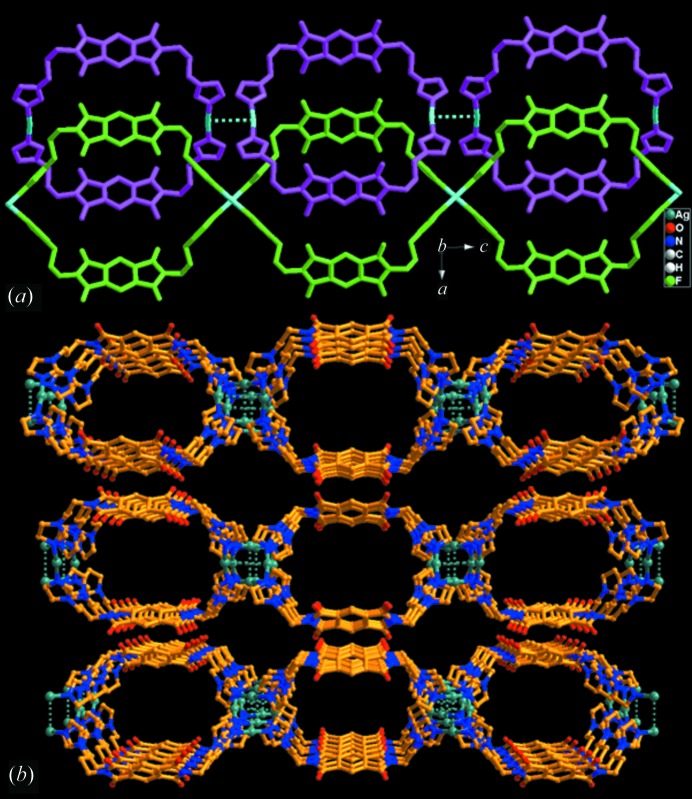
The compatible packing arrangements of the two kinds of structural motifs: (*a*) the ribbon of rings and the twisted double chains in the structure of form **A**: (*b*) the tubular channels running along the *a* axis formed by alternate packing of rings and chains in a parallel fashion in form **A**.

Crucially, the crystal packing in the structures of forms **A** and **B** displays generally the same architecture, as seen from Figs. 5 ▶ and S5. If two neighbouring Ag_2_ *L*_2_ rings in form **A** are considered to be connected *via* Ag⋯Ag (3.6 Å) interactions as shown in Fig. 5 ▶(*a*), an extended ribbon of rings is formed along the *c* axis. Such a ribbon is arranged parallel to the (Ag*L*)_∞_ twisted double chain. Furthermore, the one-dimensional ring ribbons and double chains alternately stack along the *a* axis, resulting in tubular channels made up of [Ag_2_ *L*_2_]^2+^ rings (belonging to ring ribbons) and [(Ag_1/2_)_4_ *L*_2_]^2+^ metallacycles (belonging to double chains), which are alternately overlapped (Fig. 5 ▶ *b*). The compatible crystal packing arrangements of the Ag_2_ *L*_2_ rings and the double (Ag*L*)_∞_ chains provides the circumstances for the observed isomerism and disorder to occur in the crystalline state.

**Figure 6:**
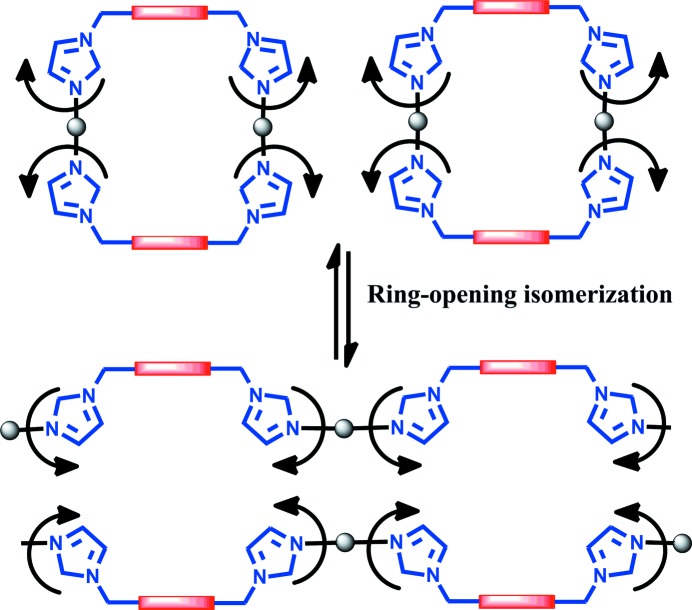
Schematic representation of the ring-opening isomerization process *via* rotation of the methylene imidazole donors and shift of the Ag centres, resulting in interconversion between discrete rings and polymeric double chains.

Insight into the disorder and crystal polymorphism offers an opportunity to realise how structural transformations between these two kinds of structural motifs could happen during the crystal growth process, which usually occurs in solution but is now manifested directly in the solid state. From the above discussion we realise that since the overall packing of the coordination skeleton frameworks can be kept static while allowing free rotation of the imidazole ring donors about the flexible propylene joints, structural conversion between Ag_2_ *L*_2_ rings and (Ag*L*)_∞_ double chains can readily take place, with the N_imino_ donors turning from perpendicular to parallel with respect to the central pyromellitic diimide base, and *vice versa*. As demonstrated in Fig. 6 ▶, such rotations can enable ring-opening isomerization between the closed rings and the extended chains (*e.g.* Su *et al.*, 2003 ▶; Zhang *et al.*, 2010 ▶). In the solid state, such ring-opening isomerization has to be accompanied by a shift of the Ag centres, while in solution such structural transformation can proceed more easily, especially in the presence of extra Ag^+^ ions (*see below*). During the structural conversion, the central pyromellitic diimide bases retain the same *syn* conformation and the Ag centres keep the same linear coordination, so the ring-opening isomerization mediated by rotation of imidazole rings is subject to only minimal energy barriers. This probably accounts for the observations of the highly variable disorder of the crystallized cyclic or polymeric isomers under very similar reaction conditions.

**Figure 7:**
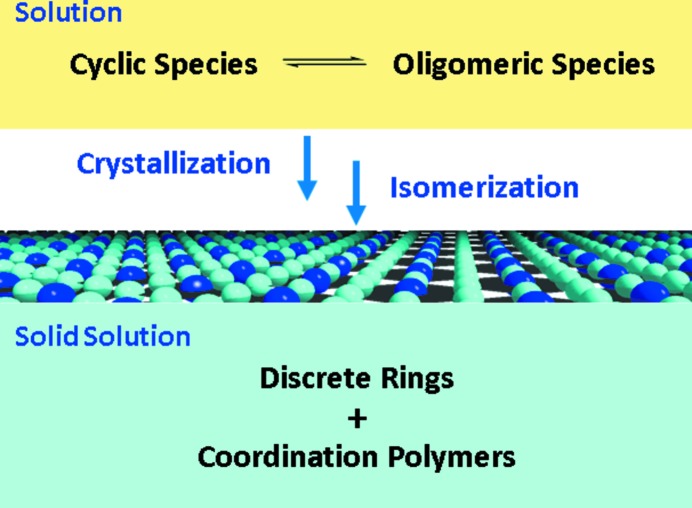
Representation of a potential crystallization process in which solid solutions containing discrete rings and coordination polymers of varying ratios are formed from equilibrium solution species *via* a ring-opening isomerization mechanism.

The structural transformation speculated above on the basis of the solid-state observations may represent a possible scenario during real crystal growth. One unique feature of the present system is that the crystal polymorphism intrinsically relies on a ring-opening isomerization process. The observed order/disorder phenomenon reflects a highly variable ratio of cyclic and polymeric isomers within one crystal. Therefore, these crystals can be regarded as solid solutions (Yeung *et al.*, 2013 ▶) containing varying amounts of the discrete Ag_2_ *L*_2_ rings and the polymeric (Ag*L*)_∞_ chains, as well as the likely presence of finite (Ag*L*)*_n_* oligomers, which is distinct from normal crystal polymorphism and isomerism phenomena (Makal *et al.*, 2011 ▶; Jiang, Li *et al.*, 2010 ▶; Stahly, 2007 ▶; Jiang, Pan *et al.*, 2010 ▶; Blagden & Davey, 2003 ▶). Formation of such solid solutions may be hypothesized, as illustrated in Fig. 7 ▶, to undergo concurrent crystallization and isomerization processes from the solution. In principle, a ‘molecular library’ (Leininger *et al.*, 2000 ▶; Lehn & Eliseev, 2001 ▶) involving discrete cyclic and oligomeric species (Yue *et al.*, 2005 ▶; Masciocchi *et al.*, 1998 ▶; Carlucci *et al.*, 1998 ▶) may be expected to exist in the reaction solution. During crystallization, this mixture may converge to either discrete rings or polymeric structures, depending on the thermodynamic or kinetic contributions or the presence of specific external influences, such as intermolecular forces, concentration and template effects. Two pathways may be speculated: one is polymerization of the ring-opened species to produce coordination polymers, or reversely, cyclization of the intermediate oligomers to produce discrete rings. In both cases, ring-opening isomerization has to occur. When the ring-opening isomerization is able to take place in a bidirectional way under appropriate conditions, both cyclic and polymeric isomers may co-crystallize in the same crystal, as in the present case (Wheaton *et al.*, 2006 ▶). Therefore, we believe that the present example may provide a unique case to shed light on the isomerization process taking place at the crystal surface, namely, structural transformation happening at the interface of the solution and solid during crystallization. Specifically, it is likely that the aromatic stacking forces may direct alignment of the solution precursors on the surface and subsequently enable the formation of either discrete rings or extended chains *via* ring-opening isomerization. Both cyclic and oligomeric species can act as precursors, and the interconversion between these species may be facile due to a low energy barrier for imidazole ring rotation. Usually the cyclic precursors are thermodynamically favoured in solution; however, formation of the chain structure in the present case may be aided by the argentophilic interactions between the double chains.

### 3.3. Solution studies   

**Figure 8:**
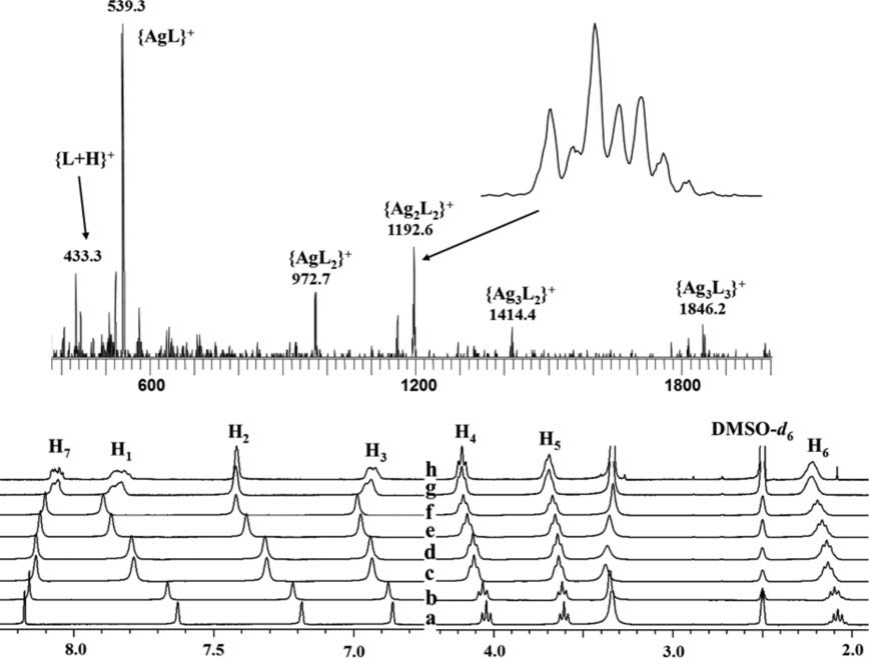
Partial ESI-MS spectrum of **1** (upper) and ^1^H NMR monitoring (lower) of the titration of AgCF_3_CO_2_ by ligand *L* with varying *M*:*L* ratios: (*a*) pure *L*, (*b*) 1:4, (*c*) 1:2, (*d*) 2:3, (*e*) 1:1, (*f*) 2:1, (*g*) 3:1 and (*h*) 4:1 (∼ 90 m*M*).

To obtain evidence to support the above hypothesis, solution structure investigations were carried out by means of electrospray ionization mass spectrometry (ESI-MS) and ^1^H NMR. The ESI-MS spectra of **1** in DMSO or DMF display major peaks, corresponding to [Ag*L*]^+^, [Ag*L*_2_]^+^, [Ag_2_ *L*_2_(CF_3_CO_2_)]^+^, [Ag_3_ *L*_2_(CF_3_CO_2_)_2_]^+^ and [Ag_3_ *L*_3_(CF_3_CO_2_)_2_]^+^ species, as shown in Fig. 8 ▶. Each assignment is verified by the isotopic patterns. Further evidence for a molecular library in solution is obtained from the ^1^H NMR measurements, which show only one set of proton signals in which all peaks are broadened and shifted relative to those of the ‘free’ ligand. This clearly suggests complexation and rapid metal–ligand exchange on the NMR timescale, consistent with dynamic equilibrium between cyclic and oligomeric species in solution.

To explore the possible distribution of the solution species, the titration of AgCF_3_CO_2_ with ligand *L* was monitored by *in situ* NMR measurements. As shown in Fig. 8 ▶, when the *M*:*L* ratio is increased from 1:4 to 4:1, a general downfield increase in the proton shift (except that of H_7_, *see below*) relative to the free ligand is observed. Detailed examination reveals that the signal shifts of these aromatic protons show three distinct steps: *L*:*M* = 2:1 and 3:2 (Figs. 8 ▶ *c* and *d*), 1:1 and 1:2 (Figs. 8 ▶ *e* and *f*), and 1:3 and 1:4 (Figs. 8 ▶ *g* and *h*). This suggests that in the situation where the ligands are in excess, two- or three-coordinated species such as Ag*L*_2_ may predominate in solution. Conversely, when the *L*:*M* ratio is closer to or exceeds 1:1, the (Ag*L*)*_n_* oligomeric species may predominate in solution. A slight excess of Ag^+^ in solution may be able to facilitate the isomerization process between cyclic and oligomeric species because the structural interconversion involves not only the rotation of the imidazole rings, but also migration of the Ag^+^ ions. In the presence of additional Ag^+^, such migration may no longer be necessary. On the other hand, when a large excess of Ag^+^ is added (*L*:*M* = 1:3 and 1:4), all N donors as well as carbonyl groups (C=O) may be over-surrounded by Ag^+^ ions, which will hinder metal–ligand exchange and cause additional structural changes, thus giving rise to significantly broadened signals.

A striking finding is that while most of the protons are shifted downfield due to a loss of electron density upon coordination (Su, Cai *et al.*, 2003 ▶), the signal of H_7_, which is located on the central pyromellitic diimide base, is shifted slightly upfield. This hints at the presence of aromatic interactions in the solution similar to the aromatic stacking observed in the solid state. The staggered stacking of pyromellitic diimide in parallel will lead to edge-to-face interactions for H_7_, thereby exposing this proton to a ring current shielding effect from the neighbouring molecule (Shetty *et al.*, 1996 ▶; Adams *et al.*, 1999 ▶; Pang *et al.*, 1999 ▶; Zvyagin, 1988 ▶). By contrast, the protons on the propylene and imidazole fragment are only subject to electron-withdrawing effects owing to complexation.

### 3.4. Phase transition in the solid state   

**Figure 9:**
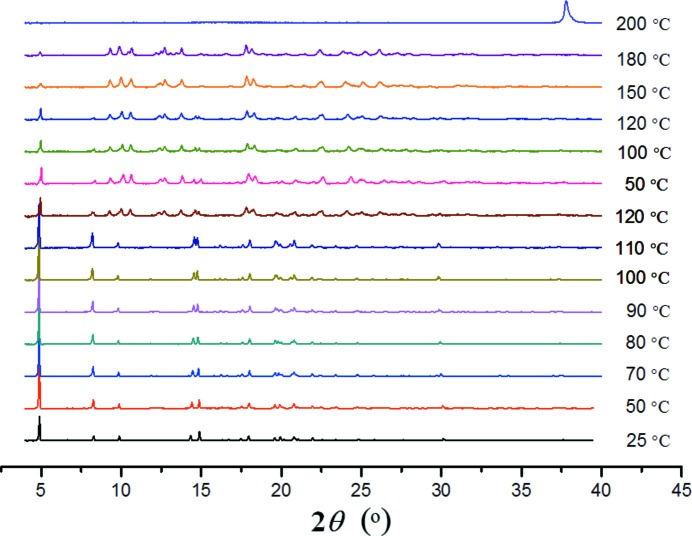
Variable-temperature powder XRD measurements monitoring the phase transitions: the bulk sample was first heated from room temperature to 120°C, then cooled to 50°C, then heated again to 200°C.

**Figure 10:**
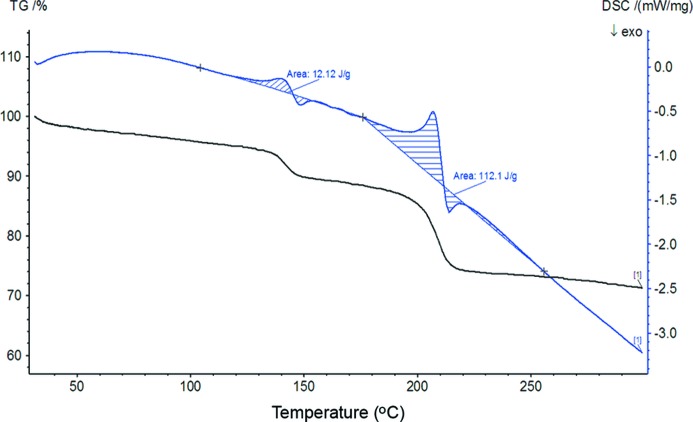
DSC (blue) and TGA (black) curves for complex **1**.

Since complex **1** displays the order/disorder phenomenon in the solid state, and structural transformation between the cyclic and polymeric isomers could take place *via* ring-opening isomerization, it might be expected that heating of the crystals could cause further phase transitions in the solid state. Fig. 9 ▶ illustrates variable-temperature powder XRD patterns recorded upon heating and then cooling of the bulk crystals, and Fig. 10 ▶ shows differential scanning calorimetry–thermogravimetric analysis (DSC–TGA) curves recorded in the range 25–300°C. From room temperature up to ∼ 110°C, there is a continuous initial weight loss indicative of gradual solvent escape, but the crystal phase remains intact. From ∼ 120°C, the XRD pattern starts to show new diffraction peaks, indicating the appearance of a new crystalline phase. This structural change may be triggered by a relatively abrupt solvent loss as seen from the TGA curve. The transition is irreversible, because the new diffraction pattern was retained when the sample was cooled back to 50°C. Elevating the temperature again from 50 to 250°C revealed that the first phase transition starting around 120°C was completed by ∼ 150°C, where the XRD pattern of complex **1** has been converted to a completely new one and the weight loss reached a stable region on the TGA curve. This new phase is stable up to 180°C and then a drastic structural change occurs around 200°C. The XRD pattern loses all diffraction peaks corresponding to complex **1** and the TGA curve displays a steep weight loss, indicating that the compound becomes principally amorphous, accompanied by evacuation of all remaining solvent from the crystal lattice around 200°C. During these two structural changes (the first crystal-to-crystal phase transition and the second crystal-to-amorphous phase conversion), the coordination framework of complex **1** may not fall apart because the DSC measurement verified that both transitions are endothermic, corresponding to solvent evaporation processes. Decomposition of the framework is observed later, in the temperature region from 300 to 650°C (Fig. S2). Attempts to collect single-crystal diffraction data for the new crystal phase around 120°C were not successful, since the crystallinity became poor upon slow heating. A possible reason may be that upon elevating the temperature and releasing the solvent, rotation of the imidazole rings and migration of Ag are promoted in the solid solutions. This would trigger structural transformation between the cyclic and polymeric isomers, although the overall packing arrangement of the ring/chain frameworks remains almost unchanged, thus leading to a crystal-to-crystal phase change. However, since the phase transition is mediated by random movements of the imidazole rings and Ag in the solid state, the disorder along the *a* axis is likely to become more severe upon heating, thus affording unsatisfactory Bragg reflections and diffraction patterns. In the second structural change around 200°C, the structure may become completely disordered, so as to destroy the three-dimensional crystal lattice.

## 4. Conclusions   

In summary, we have described a rare example in which Ag_2_ *L*_2_ ring and (Ag*L*)_∞_ chain motifs co-crystallize in varying ratios in the same crystal, showing variable degrees of disorder depending delicately on the crystallization conditions. The structural analysis reveals a straightforward pathway for transformation between the cyclic and polymeric isomers, which can proceed through ring-opening isomerization by facile rotation of the ligand donor groups. Solution structural investigations suggest the existence of a dynamic equilibrium between cyclic and oligomeric species, with aromatic stacking interactions occurring amongst the solution species. Therefore, the crystallization process may be influenced by preferential aromatic stacking of the bulky ligands to direct alignment of coordination precursors on the crystal surface, followed by crystal growth involving ring-opening isomerization at the interface between the solution and the solid. Such ring-opening structural transformation can apparently also occur in the solid state upon heating, which may be the origin of an observed transition between crystalline phases. In general, this chemical system may offer a chance to observe directly ring-opening isomerization at the crystal surface, providing a prototypical model system for understanding of the ring-opening polymerization process, which is usually limited to indirect study of solution systems.

## Supplementary Material

Crystal structure: contains datablock(s) A_Pbcm, B_Ibam. DOI: 10.1107/S2052252514015966/bi5033sup1.cif

Structure factors: contains datablock(s) A_Pbcm. DOI: 10.1107/S2052252514015966/bi5033A_Pbcmsup2.fcf

Structure factors: contains datablock(s) B_ibam. DOI: 10.1107/S2052252514015966/bi5033B_ibamsup3.fcf

Idealized CIF for alternating ring and chain structure. DOI: 10.1107/S2052252514015966/bi5033sup4.txt

Idealized CIF for chain structure. DOI: 10.1107/S2052252514015966/bi5033sup5.txt

Idealized CIF for ring structure. DOI: 10.1107/S2052252514015966/bi5033sup6.txt

Extra tables and figures. DOI: 10.1107/S2052252514015966/bi5033sup7.pdf

CCDC references: 996604, 996605

## Figures and Tables

**Table 1:** Crystallographic data for complex **1** in crystal forms **A** and **B**

	Form **A**	Form **B**
Crystal data		
Chemical formula	C_44_H_40_Ag_2_N_12_O_8_	C_24_H_20_AgF_3_N_6_O_11_
*M*_r_	1080.62	733.33
Crystal system, space group	Orthorhombic, *Pbcm*	Orthorhombic, *Ibam*
*a*, *b*, *c* (Å)	12.6641 (14), 26.230 (3), 35.269 (4)	6.4005 (6), 26.426 (3), 35.016 (3)
*V* (Å^3^)	11715 (2)	5922.5 (10)
μ (mm^−1^)	0.72	0.77
Crystal size (mm)	0.40 × 0.40 × 0.16	0.33 × 0.15 × 0.05
		
Data collection		
*T*_min_, *T*_max_	0.757, 0.891	0.866, 0.953
No. of measured, independent and observed [*I* > 2σ(*I*)] reflections	57 799, 11 087, 4492	14 814, 2801, 1812
*R*_int_	0.095	0.050
(sin θ/λ)_max_ (Å^−1^)	0.606	0.606
		
Refinement		
*R*[*F* ^2^> 2σ(*F* ^2^)], *wR*(*F* ^2^), *S*	0.101, 0.233, 1.02	0.091, 0.199, 1.12
No. of reflections	11 087	2801
No. of parameters	621	377
No. of restraints	84	270
Δρ_max_, Δρ_min_ (e Å^−3^)	1.28, −1.52	0.79, −0.51
