# Supplementary material for: Structural disorder and transformation in crystal growth: direct observation of ring-opening isomerization in a metal–organic solid solution
Source: IUCrJ. 2014 Aug 20;1(Pt 5):318–27. doi: 10.1107/S2052252514015966 (PMC4174874; doi:10.1107/S2052252514015966)

# IUCrJ

**Volume 1 (2014)**


**Supporting information for article:**

## Structural disorder and transformation in crystal growth: direct observation of ring-opening isomerization in a metal–organic solid solution

**Ji-Jun Jiang, Jian-Rong He, Xing-Qiang Lü, Da-Wei Wang, Guo-Bi Li and Cheng-Yong Su**

# Supporting information

**Table S1** Selected geometric parameters (Å, º)

| | | | |
|---|---|---|---|
| **Form A** | | | |
| Ag1—N4 | 2.053 (8) | Ag2—N10 | 2.121 (9) |
| Ag1—N1 | 2.087 (10) | Ag2—N7 | 2.124 (8) |
| Ag1—Ag1$^{i}$ | 3.0776 (17) | Ag1'—Ag1'$^{i}$ | 3.031 (9) |
| N4—Ag1—N1 | 176.6 (4) | N1—Ag1—Ag1$^{i}$ | 92.6 (3) |
| N4—Ag1—Ag1$^{i}$ | 90.7 (2) | N10—Ag2—N7 | 171.7 (3) |
| **Form B** | | | |
| Ag1—N11$^{ii}$ | 2.134 (7) | Ag2—N11$^{iii}$ | 2.130 (7) |
| Ag1—Ag1$^{iii}$ | 3.086 (3) | Ag2—Ag2$^{ii}$ | 3.645 (4) |
| N11—Ag1—N11$^{ii}$ | 178.2 (4) | N11—Ag2—N11$^{iii}$ | 167.8 (4) |

Symmetry code(s): (i) *x*, −*y*+1/2, −*z*; (ii) −*x*+2, *y*, −*z*+1/2; (iii) −*x*+2, −*y*+1, *z*.

**Figure S1** IR spectrum of complex **1**.

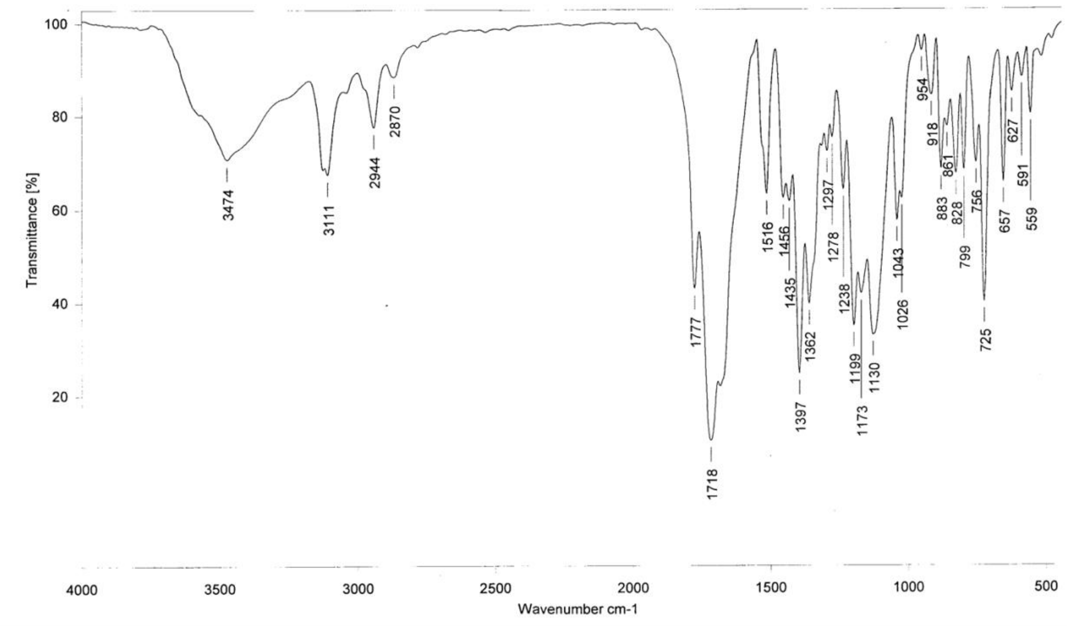


**Figure S2** TGA curve of complex **1** up to 700°C

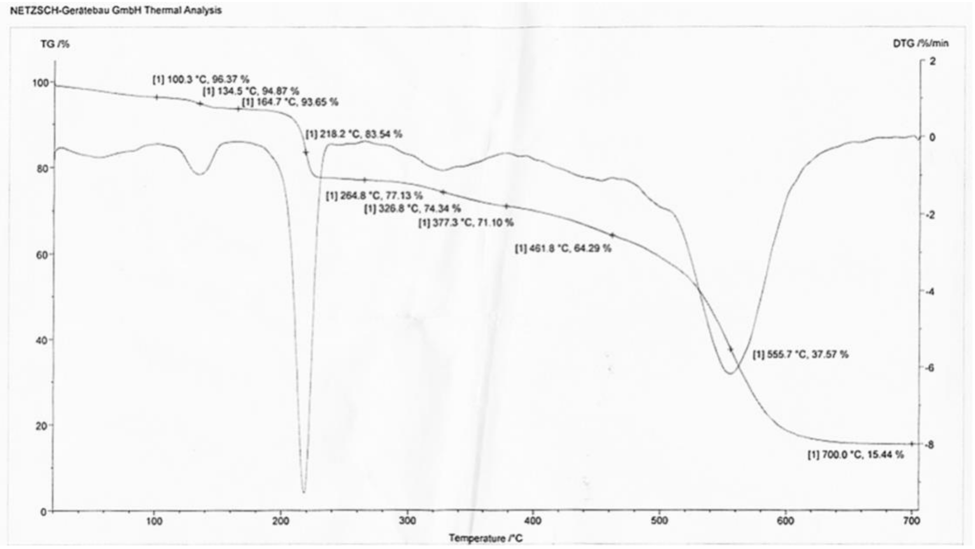

**Figure S3** Excitation and emission spectra of the ligand *L* and complex **1** in the solid state.

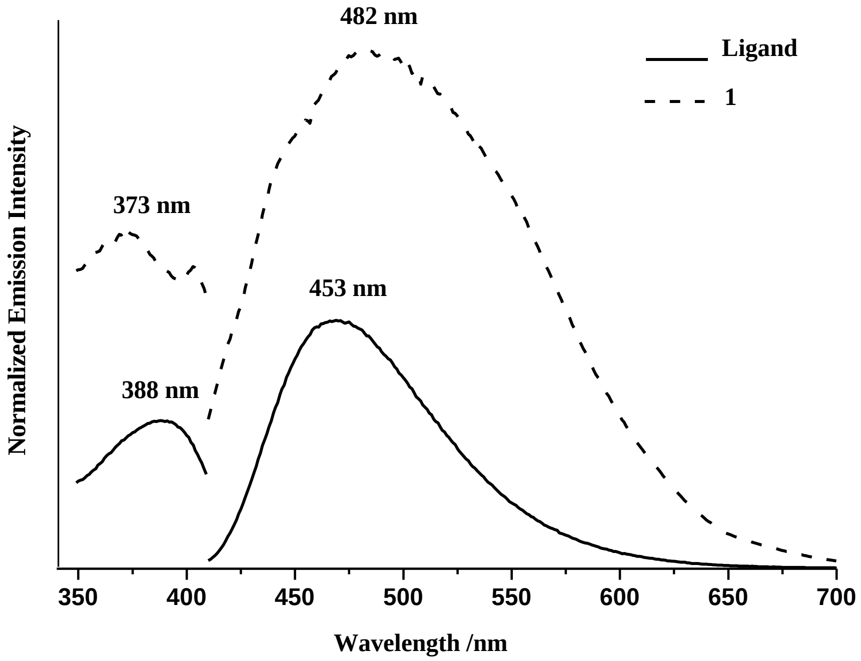


**Figure S4** Views of the twist double chain in **1** in the space-filling mode from two different directions.

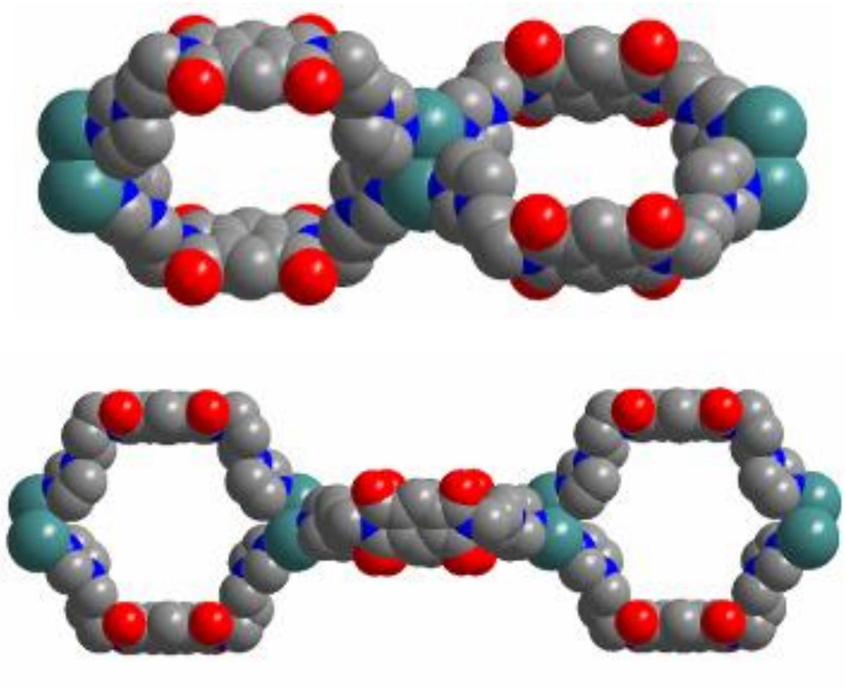

**Figure S5** The unique packing arrangement of two types of structural motifs in **1**: (a) parallel stacking of the ribbons of rings and the twist double chains in Form **A**, and 50-50% distribution in Form **B**, (b) tubular channels formed by the overlapping arrangement of two types of structural motifs in the *a* direction showing counter anions and solvent molecules in Form **B**, and (c) space-filling representations of the tubular framework without counter anions and solvent molecules in both Form **A** and **B**.

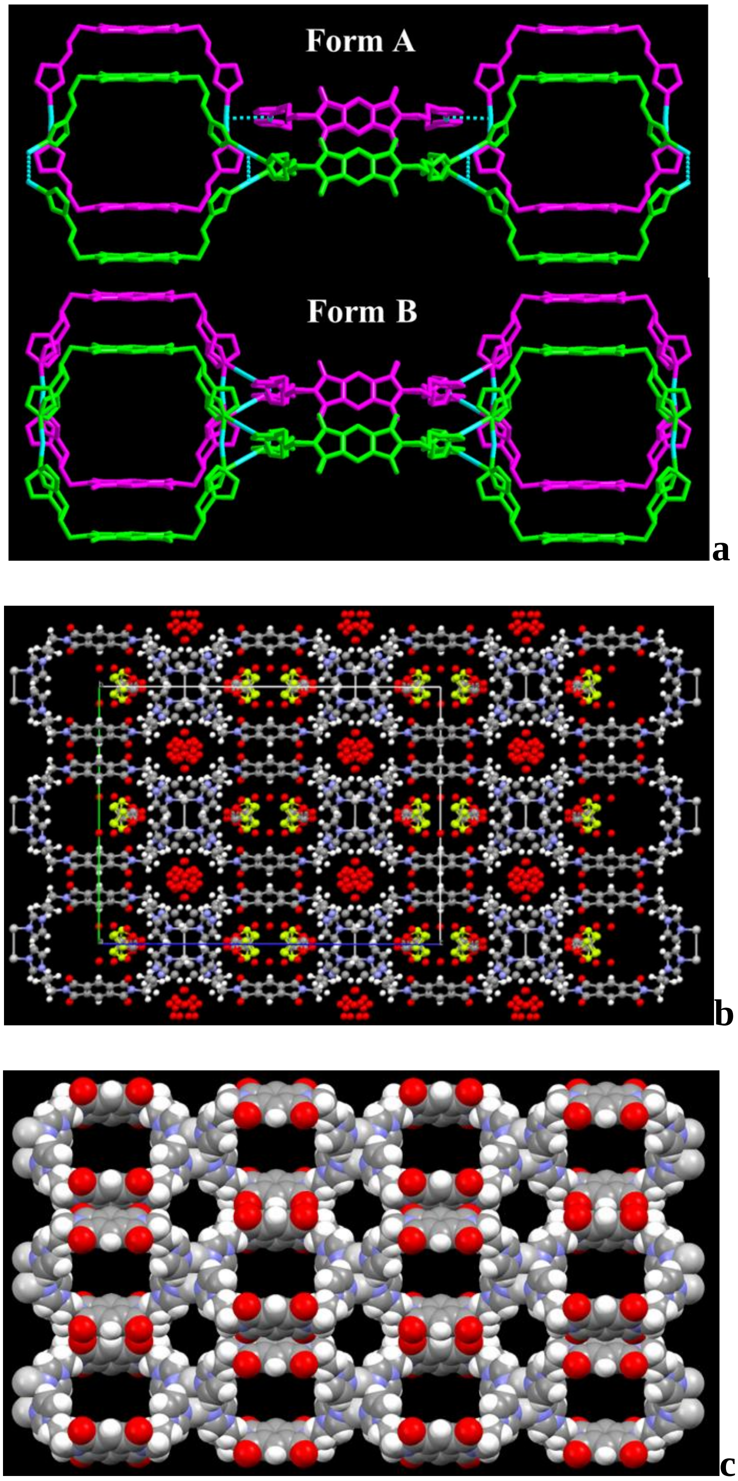

Supplement: Supplementary file 7 [file m-01-00318-sup7.pdf]
